# Medical Opinion and Movement

**Published:** 1909-06-05

**Authors:** 


### Medical Opinion and Movement.

THERE is a very definite idea among the laity, and to some extent also among the medical profession, that accidents or sudden emotions sustained during Pregnancy may have a prejudicial effect upon the development of the fœtus, and may be responsible for arrests in development and malformations. The report of Dr. S. Rebaudi, therefore, upon 25 pregnant women who escaped from the Messina earthquake is not without interest. Of these 25 women one was confined prematurely at the seventh month, but she sustained a severe abdominal injury. Thrown down by some falling debris, she remained for three days with a heavy stone pressing upon the abdomen. Almost immediately afterwards she was delivered of a stillborn child. One other patient aborted a month and a half after the earthquake, but apparently this was due to an endometritis. All the others went to full term and were delivered of children in all respects healthy and normal. All these women, however, endured terrible physical and moral sufferings. Flight during the night, exposure to cold, grief for lost relatives, insufficient food and drink, and varying kinds of injuries, one patient sustaining a fracture of the leg. The author states that some of these women were extremely nervous and had previously experienced miscarriages. It would appear, therefore, from these experiences that only traumatic injuries directly or indirectly affecting the uterus are able to influence the course of pregnancy.

---

DR. G. J. MULLER, of Berlin, reports beneficial results from the administration of thiosinamine and sodium salicylate (fibrolysin) by intra-muscular injections in cases of Tabes Dorsalis. He happened to observe the effects of this drug upon the disease, although it was given for a different purpose. A tabetic patient was afflicted with paralysis of the bladder complicated with an indurated urethral stricture, dilatation of which presented considerable difficulty. Dr. Müller had resort to fibrolysin injections with the view of influencing the stricture, and to his surprise the injections were followed by amelioration of the vesical crises and complete disappearance of the lightning pains in the lower limbs. Since then he has tried these injections in eleven other cases of tabes. Of these, five have completely lost their lightning pains, and the other six, who are still under treatment, have so far improved that they have completely recovered their sleep. In one case severe gastric crises have disappeared, in another the same effect has been produced upon vesical and rectal crises, and, in a third, there was considerable improvement in laryngeal crises. The bladder has recovered its power in several cases, and micturition has become much easier. An improvement was also observed in the general condition, but this may be due to the absence of the pains and the return of sleep. No improvement was observed in the ataxia and other objective symptoms of the disease. The author injected every other day or every day one cubic centimetre of the following solution: Thiosinamine, 10 grammes; glycerine, 10 grammes; sodium salicylate, 20 grammes; and aqua distill. ad 100 grammes. No phenomena of intoxication or other ill effects followed the injections.

---

DR. MAYGRIER, of "La Charité," claims special success in the Treatment of Eclampsia. With 37 cases during the last three years at this hospital, he has only had one death, giving a mortality of 2.7 per cent. The chief therapeutic means upon which he relies is Venesection. He recognises three chief indications for treatment: The patient is in a condition of intoxication, and means must be adopted to get rid as far as possible of the poison, arterial tension is raised and endeavours must be made to reduce it, and the kidneys are working inefficiently or not at all, and renal secretion must therefore be re-established. The first two indications are best met by venesection; but it must be thorough and abundant, to the extent of 1,000 grammes or more. As soon as a patient is admitted into the hospital and recognised to be eclamptic, she is bled. The amount varies according to conditions, but a fall of at least 10 or 12 centimetres of mercury is aimed at. The bleeding should be slow, lasting 20 to 30 minutes, and it should be repeated if the blood-pressure rises again or if there is a reappearance of the fits. After venesection a purgative (generally seidlitz powders) is administered, if necessary by the stomach-tube, and then rectal lavage of 20 to 30 litres. In cases of severe headache and repeated fits lumbar puncture may be resorted to. Every two or four hours a catheter is passed in order to control the progress of renal secretion. The basis of the treatment, however, is liberal venesection. In most cases, according to Dr. Maygrier, the fits cease after the first thorough bleeding; but if they recur, a second venesection suppresses them altogether.

---

IN an interesting article in the *American Journal of Surgery* Dr. Henry Mann Silver, of New York, draws attention to the surgical importance of a proper recognition of the visceral crises in the erythema group of skin diseases. Although these conditions are by no means common, failure to recognise them may lead to a faulty diagnosis of some abdominal lesion such as appendicitis or intussusception and consequent unnecessary surgical interference. To Professor Osler more than any other writer credit is due for calling the attention of the profession to this possible source of error, and he has collected 29 cases illustrating the subject. These cases may be divided into three chief groups: Quincke's disease, in which there is colic associated with angio-neurotic œdema; Henoch's purpura, in which there is arthritis combined with erythema or purpura and colic; and the third group consisting of recurring attacks of colic without the appearance of any skin lesion for a prolonged period, even years. Dr. Mann Silver reports several interesting cases in which operation has either been performed, or has only just been avoided by the timely development of an arthritis or a skin lesion. In children, especially, with colic, the greatest care should be taken to get a full history, which may bring out the fact of previous attacks, either of skin lesions or arthritis or of intestinal crises, and a careful examination of the skin should be made for angio-neurotic œdema, purpura, or erythema. A differential blood count may also help in the diagnosis. In Henoch's purpura there is slight leucocytosis and a normal differential count. In intussusception and appendicitis there is a leucocytosis with increase in the polymorphonuclear cells.

---

At a recent meeting of the New York State Medical Society, Dr. Whitman, whose name is well known in connection with orthopædic surgery, read a paper on his results in the treatment of cases of Fracture of the Neck of the Femur by Abduction. This, which is known on the Continent as the Morisani method, has already been very favourably reported upon by Bardenheuer of Cologne, and we believe we are correct in stating that it is the favourite method in use in German clinics. In England, notwithstanding the enthusiastic advocacy of Professor Ralph Thompson in last year's Hunterian Lectures, the method seems to be practically unknown. Whitman, in discussing the method, points out that hitherto the teaching with regard to the infrequency of perfect functional recovery in femoral neck fractures has been bolstered up by four main arguments: that the deficient blood supply explains non-union, even if the fragments are opposed; that these fractures are often the result of great violence, producing much shattering of bone; that correction of deformity, if the fragments are impacted, must not be undertaken; and, finally, that disorders of nutrition leading to bony atrophy often follow the injury. None of these objections holds in the majority of cases, and the author sums up the proper principles of treatment as follows: Immediate and complete reduction of deformity, no matter to what it may be due, and secure fixation until there is no longer any question of displacement. These conditions are often very difficult to obtain in the hip joint, and the author considers the abduction method gives the most satisfactory results in the majority of cases.

---

It may be worth while here to describe the Morisani method as modified by Dr. Whitman: "The patient having been anæsthetised, the upper part of the trunk is placed on a box. The sacrum rests on a secure pelvic support, and each lower limb is held by an assistant in the extended position, so that the body is symmetrical, the pelvis level and perfectly balanced. The operator stands on the injured side, his hands supporting the thigh. The assistant, holding the sound limb in the extended position, abducts it to the normal limit, which is reached when the outer border of the neck comes into contact with the upper rim of the acetabulum. If the limb be held firmly in this position, the pelvis is fixed, the anterior superior spines lying in the same plane. At this moment the assistant, supporting the injured limb in slight flexion, rotates it inward to the normal attitude; then, under steady traction, abducts it slowly, the operator meanwhile supporting and guiding the thigh and pressing downward on the trochanter, which should regain the normal relation to Nelaton's lines, when the deformity, under the combined influence of pressure, leverage, and traction, is corrected. When the limb has been abducted to the desired degree—preferably the normal limit as indicated by comparison with its fellow—a firm, well-fitting plaster spica is applied from the upper part of the thorax to the toes." In conclusion, the author gives the following warning: "The abduction treatment is not automatic. It must be used with discretion, and its proper application may require more skill and experience than the ordinary methods of routine. It simply enables one, as it were, to lay the foundation of success, which, as experience has now demonstrated, is no longer beyond the scope of surgical endeavour."

---

Dr. Crile's interesting monograph on Hæmorrhage and Transfusion, which has lately been published by Appletons, will be of interest to both physicians and surgeons, and its practical value is unquestioned. Briefly stated, the author has reported an extensive series of experimental studies on the phenomena of hæmorrhage in man and the lower vertebrates, and the treatment of this condition, experimentally and clinically considered. Of all American surgeons, Dr. Crile has, perhaps, been the most enthusiastically energetic in testing his experimental results at the bedside—the only real and conclusive test of the practical value of laboratory methods—and his findings will therefore appeal to a large section whom mere laboratory conclusions may leave cold. Specially important in this connection is the careful elaboration of transfusion methods, and the sane comparison of their clinical value. Those interested in the more theoretical study of tumour relations will find the chapters dealing with the work of Beebe, who noted remarkable retrogressions in animal tumours in cases experimentally transfused with normal blood, of much value, and especially so since negative as well as positive findings are equally faithfully set down. Crile has found transfusion of decided benefit in cases of hæmorrhage resulting from intestinal ulceration or operative interference, and the description of the clinical method used is particularly valuable owing to its clearness. The method is being tried in London hospitals, but is worthy of a much more general trial, and, reading Dr. Crile's cases, it is evident that there may arise many occasions when a knowledge of the benefits to be derived from transfusion would be of practical importance in private practice.

---

Among recent papers dealing with the Diagnosis and Treatment of Appendicitis—matters which must always be of importance to the practitioner, especially in these days when so many differences of opinion exist as to the exact time when surgical interference is desirable—two in the *Boston Medical and Surgical Journal* are particularly commendable because of the clearness and brevity with which the authors treat their themes. Beverley Robinson, discussing the treatment, remarks that it is often very difficult to diagnose between cases of pure appendicitis and colitis. When an absolute diagnosis of appendicular trouble is impossible he recommends expectant treatment—rest in bed, hot-water bag applications to the abdomen, laxative enemata, and two hourly doses of codeine in very small amounts from $\frac{1}{20}$ to $\frac{1}{5}$ of a grain by mouth if the pain requires it. Hypodermic injections of morphine are not advisable. The author has found 10-grain doses of salicin, given four hourly, of distinct use in treating such cases, and makes it a rule to limit the diet to fluid, given in very small quantities. The indications for operative interference are not distinctly laid down, and the author appears to look unkindly on early operations, remarking that " very many cases of perforation cause an abscess limited by protective false membrane, if not operated on too soon or ill-advisedly "—a statement which is hard to prove, and the truth of which it would be manifestly ill-advised for the practitioner to take for granted in every case. Fisher, in an article dealing more particularly with the differential diagnosis of appendicitis, states that in 51 per cent. of cases the appendix can be felt on palpation—another statement which is open to grave doubts, although it is qualified by the condition that the organ must lie upon the aponeurosis of the psoas, which must be relaxed by flexion of the thigh, and that the abdominal walls must not be rigid. McBurney's point, as Lauz has proved, does not correspond to the situation of the appendix, but Fisher believes that tenderness over this point is of great diagnostic importance.

---

SELIG, of Frauzenbad, in a paper read before the Medical Society of Prague, summarises a series of interesting observations on the heart of sportsmen. Many authorities have alleged, on evidence obtained by percussion, auscultation, and cardio-graphic tracings, that an inevitable concomitant of strenuous exercise is a dilatation of the heart. Some of them have even attempted to show that moderate exercise brings about such dilatation. Mendel and Selig have employed orthodiagraphy and orthodiaphanoscopy in investigating this question, and have found, as has already been alleged by Lennhoff and Levy-Dorn, that even in professional wrestlers there is no evidence that excessive muscular exercise leads to cardiac dilatation. Now the authors have gone a step further, and have investigated the hearts of professional swimmers immediately after long-distance races. They found that in ten cases out of eleven there was diminution of, instead of an increase in, the volume of the heart, a fact which they explained by the general dilatation of the blood vessels which is known to follow muscular exercise. In a large number of professional cylists, Marathon racers, and football players they were unable to prove any dilatation of the heart immediately after a bout of violent exercise. They conclude for these findings that although cardiac lesions are not rare amongst amateur sportsmen, they are by no means common in trained professionals, and that Bryers statement that in Austria alone more than 250,000 men have been incapacitated from service in the army owing to excessive muscular exercise as bicyclists, football players, etc., is absolutely unfounded.

WHEREAS Angina, or painful difficulty in swallowing and breathing, is for the most part treated by the buccal route, Berliner for ætiological reasons proposes to apply treatment to the nose. In the *Münchener Medizinsche Wochenschrifte* he describes a method whereby the patient makes applications to the nasal mucous membrane of small quantities of an ointment consisting of protargol 1½ grams dissolved in 2½ grams of distilled water, to which is added 6 grams of anhydrous lanoline, .1 grams of menthol, 3 grams of saccharine, and sufficient vaseline to bring the total weight up to 15 grams. The menthol is used because of its effect on congested surfaces, the saccharine to improve the taste of the mixture. As soon as the flavour of the menthol has disappeared from the patient's pharynx, to which it travels quickly, the ointment must be applied again. The author claims to have had favourable results with this treatment in cases of catarrhal angina, as well as upon the cold which accompanies this affection. Parenchymatous angina has cleared up yet more quickly than the last variety, even when accompanied by an abscess which needed opening. All varieties of the effection which are accompanied by an exudation are rapidly cleansed, with the exception of angina due to syphilis, which needs the application of anti-syphilitic remedies. The treatment is exceedingly useful as an adjuvant to antitoxin in cases of diphtheria of the nose and throat. The author has also used the ointment when treating acute coryza.

---

KIRCHNER, in the *Deutsche Zeitsch F. Chirurgerie*, describes two cases of Traumatic Motor Aphasia treated by operation with successful results. In both cases the condition was the result of a head injury. In the first case, that of a man of 53, the patient after the accident could only say " Yes " and " No "; he was unable to repeat words or to read them aloud, but was able to understand what was said to him, to write and to read. At the operation a splinter of bone 6 centimetres long was found lying on the left inferior frontal convolution and in the inferior part of the fissure of Rolando, without, however, penetrating the brain substance. This splinter was removed, and the following day the patient was able to talk disjointedly and pronounce words he had been unable to pronounce two days before. In about three weeks all aphasic symptoms disappeared. The second patient, aged 26, who had suffered a more violent accident, was only able to pronounce a few inarticulate words, but was able to understand orders given. A right facial paralysis was present, and the pulse was slowed. A subdural hæmatoma, 6 centimetres in extent, was removed from the same locality as the splinter in the previous case. The following day the patient was able to say " Yes " and " No," to write and to read. In a week's time the patient commenced to regain slowly his power of speech. The author classifies these cases under the heading of sub-cortical motor aphasia, holding that such a heading does not exclude cortical lesions such as were present in these cases.

